# Oral Lichen Planus and Oral Lichenoid Lesion in Pediatric Patients: A Narrative Review of Published Case Reports

**DOI:** 10.3390/reports9030277

**Published:** 2026-08-20

**Authors:** Konstantinos Poulopoulos, Christina Charisi, Filippos Fytros, Asterios Katsagkolis, Stefanos Zisis, Maria Kalyva, Nikolaos Spantidakis, Petros Papadopoulos, Athanasios Poulopoulos, Vasileios Zisis

**Affiliations:** 1 Department of Dentoalveolar Surgery, Surgical Implantology and Radiology, School of Dentistry, Aristotle University of Thessaloniki, 54124 Thessaloniki, Greece; 2 Department of Oral Medicine and Pathology, School of Dentistry, Aristotle University of Thessaloniki, 54124 Thessaloniki, Greeceakpoul@dent.auth.gr (A.P.); 3 Department of Dentistry, School of Dentistry, European University Cyprus, Nicosia 2404, Cyprusm.kalyva@euc.ac.cy (M.K.);; 4 Centre for Dental, Oral and Maxillofacial Health, School of Dentistry, 97070 Würzburg, Germany; 5 Clinic for Operative Dentistry, Periodontology and Preventive Dentistry, University Hospital RWTH Aachen, 52074 Aachen, Germany

**Keywords:** oral lichen planus, pediatric oral medicine, children, oral mucosal disease, oral pathology

## Abstract

**Background:** Oral lichen planus (OLP) is a chronic inflammatory mucocutaneous disorder that is well documented in adults but remains relatively uncommon in the pediatric population. Due to its rarity, knowledge regarding its epidemiology, etiology, clinical behavior, diagnosis, and management in children remains limited. **Objective:** To review the current literature regarding epidemiology, pathogenesis, clinical presentation, diagnosis, histopathological characteristics, treatment, and outcomes of OLP in pediatric patients. **Materials and Methods:** An electronic search of the literature was conducted in the PubMed, Scopus, and Cochrane Library databases to identify OLP-associated case reports in the pediatric population. The search was limited to English-written publications of the past decade. The initial PubMed search yielded 228 publications. Following restriction to studies published between 2016 and 2026, 97 records remained. Limiting the search to English-language publications resulted in 95 studies. Screening for patients younger than 18 years identified 51 potentially relevant publications. After title, abstract, and full-text review, 10 studies fulfilled the inclusion criteria. To ensure comprehensive literature coverage, supplementary searches were subsequently conducted in the Scopus and Cochrane Library databases, identifying three additional eligible studies after excluding the duplicates. Consequently, a total of 13 studies were included in the final review. **Results:** Pediatric OLP is considerably less common than adult disease. The available evidence suggests an immune-mediated pathogenesis, although the exact mechanism remains unclear. Clinical presentations include reticular, erosive, plaque-like, linear, and severe mucocutaneous forms, with the reticular subtype being the most frequently reported. Histopathological findings consistently demonstrate basal cell degeneration and a dense subepithelial lymphocytic infiltrate. Topical corticosteroids remain the most commonly prescribed treatment and are generally associated with favorable clinical outcomes. **Conclusions:** Although relatively uncommon, OLP should be considered in the differential diagnosis of persistent oral white lesions in children. Early diagnosis and appropriate management are essential for symptom control and prevention of complications. Additional research is needed to better understand disease pathogenesis and establish evidence-based treatment protocols for pediatric patients.

## 1. Introduction

OLP is a chronic inflammatory disorder affecting the oral mucosa and is considered part of a broader mucocutaneous disease spectrum. The condition is characterized by immune-mediated damage to the basal epithelial layer and may present in several clinical forms, including reticular, erosive, atrophic, plaque-like, papular, and bullous variants. Furthermore, oral involvement is among the most frequent manifestations, and in many patients the oral cavity is the only affected site. While OLP is relatively common in adults, it is rarely reported in children [1,2,3].

OLP is relatively common in adults, affecting approximately 0.5–2% of the general population [4]. The condition predominantly affects middle-aged and elderly individuals and demonstrates a female predilection in most epidemiological studies [4]. Interestingly, while adult cohorts show a distinct female bias, the pediatric cases captured in this review demonstrated an equal 1:1 gender distribution, suggesting that hormonal influences characteristic of adult disease may play a less prominent role in childhood presentations. In contrast, pediatric OLP is rare and accounts for only a small proportion of reported OLP cases [4]. The low incidence in children may contribute to delayed diagnosis and underreporting, particularly because many lesions are asymptomatic and are discovered only during routine dental examinations [5].

Although OLP is traditionally considered an adult disease, pediatric cases provide a unique opportunity to explore disease mechanisms because children generally have fewer confounding factors such as chronic medication use, tobacco exposure, and systemic comorbidities [6]. The rarity of pediatric OLP has limited epidemiological investigations, resulting in significant gaps regarding prevalence, risk factors, long-term prognosis, and optimal therapeutic strategies [7].

The low prevalence of pediatric OLP has resulted in limited clinical experience and a scarcity of high-quality studies. Most available evidence consists of isolated case reports and small case series [1,2,3,8,9,10,11,12]. Consequently, many aspects of pediatric OLP, including prevalence, risk factors, clinical behavior, and optimal management strategies, remain incompletely understood.

The pathogenesis of OLP is believed to involve a cell-mediated immune response directed against basal epithelial keratinocytes [13]. OLP is currently regarded as a chronic T cell-mediated inflammatory disorder characterized by apoptosis of basal keratinocytes and disruption of epithelial homeostasis. Increasing evidence suggests that both innate and adaptive immune responses contribute to disease development, involving activated CD8+ cytotoxic T lymphocytes, dendritic cells, mast cells, and multiple inflammatory cytokines, including tumor necrosis factor-α, interferon-γ, interleukin-6, and interleukin-17 [14,15,16,17]. Activated CD8+ cytotoxic T lymphocytes induce apoptosis of basal keratinocytes, leading to degeneration of the basal cell layer [13]. This process results in disruption of the epithelial-connective tissue interface and the development of chronic inflammation [13]. Several inflammatory mediators, including tumor necrosis factor-alpha (TNF-α), interferon-gamma (IFN-γ), interleukin-6 (IL-6), and interleukin-17 (IL-17), have been implicated in disease progression [18]. Recent molecular studies have also highlighted the role of oxidative stress, epithelial barrier dysfunction, and dysregulated cytokine signaling in disease pathogenesis [19,20]. Oxidative stress has been proposed to be a contributing factor in the pathogenesis of OLP [21]. This is attributed to the excessive generation of ROS and RNS in addition to the compromised antioxidant defense mechanism, which results in lipid peroxidation, protein oxidation, and DNA damage, leading to apoptosis of basal keratinocytes and inflammation [21]. In addition to this, oxidative stress triggers other pathways like NF-κB and MAPK pathways, which sustain cytokine and T cell activity and thus support immunity responses. In addition to these, any change in mitochondrial activity and cell metabolism will increase ROS, resulting in a vicious circle of oxidative stress and inflammation [21]. Increased presence of oxidative biomarkers, such as malondialdehyde (MDA), nitric oxide (NO), and 8-hydroxy-2′-deoxyguanosine (8-OHdG), along with decreased antioxidative defense, has been reported in OLP patients; therefore, oxidative stress can serve as a potential diagnostic marker [21,22].

Some recent research indicates that other than T cell mediated immunity, the humoral immune response may be involved in the development of the condition in erosive cases of OLP [23,24]. Antibodies against Desmoglein-1 (DSG1), Desmoglein-3 (DSG3), and occasionally BP180 and BP230 are found in circulation, indicating an overlapping immunoserological condition with autoimmune blistering disorders. While their pathogenic role is not yet clear, they are considered biomarkers indicative of the disease and need to be further studied in future studies [23,24].

Although the exact initiating factor remains unknown, genetic predisposition, psychological stress, infections, medications, dental restorative materials, and autoimmune dysregulation have all been proposed as contributing factors [13,18].

Recognition of OLP in children is important because the disease may mimic other oral white lesions, including oral lichenoid reactions, chronic candidiasis, lupus erythematosus, leukoplakia, and inherited keratotic disorders. Furthermore, some pediatric cases have been associated with systemic diseases, medications, genetic disorders, and environmental triggers [8,10,25,26].

OLP is currently recognized as an oral potentially malignant disorder by the World Health Organization [27]. Malignant transformation is uncommon but has been documented in adult populations [5]. While malignant transformation appears exceedingly rare in children, studies conducted in adult populations have reported a small but measurable risk of progression to oral squamous cell carcinoma, emphasizing the importance of long-term clinical surveillance. Chronic inflammation and epithelial alterations are believed to contribute to the carcinogenic process [5,16]. The risk appears to be greater in erosive and atrophic forms than in reticular lesions [5]. Although OLP is classified generally as an oral potentially malignant disorder by the World Health Organization [27], malignant transformation has not been convincingly documented within the pediatric population in historical cohorts [6,7]. Nevertheless, periodic long-term clinical follow-up remains advisable for these young patients [7].

According to the WHO consensus report led by Warnakulasuriya et al. (2021), OLP and oral lichenoid lesion (OLL) should be regarded as separate entities, rather than being merged into a single analytical category when assessing malignant potential or epidemiological outcomes [27]. The report specifically recognized that there is sufficient evidence to classify OLL as an oral potentially malignant disorder (OPMD) on its own.

Key distinctions consistent with the 2021 consensus include the following. OLP is considered a chronic immune-mediated mucosal disease with characteristic clinical and histopathological features, typically presenting as bilateral and symmetrical lesions [27]. OLL represents a heterogeneous group of lesions that may be associated with identifiable triggers such as medications, dental restorative materials, graft-versus-host disease, or other local/systemic factors. Histopathologically, OLL often demonstrates features that are less typical of classic OLP, including a deeper inflammatory infiltrate and the presence of eosinophils or plasma cells [27]. As a result, OLP and OLL should be considered distinct clinicopathological entities and should not be combined for analysis. However, given the rarity of pediatric OLP and the possibility of overlapping diagnoses in clinical practice, our methodology included both entities in an effort to discover all relevant cases in the literature.

This narrative review aimed to synthesize current evidence regarding the epidemiology, etiopathogenesis, clinical presentation, diagnosis, histopathological characteristics, treatment approaches, and outcomes of OLP and OLL in pediatric patients.

## 2. Materials and Methods

A literature search was conducted in the PubMed, Scopus, and Cochrane Library databases to identify OLP- and OLL-relevant studies published between 2016 and 2026.

The search strategy was initially developed for PubMed using MeSH terms and free-text keywords and was subsequently adapted for the Scopus and Cochrane Library databases using the corresponding database-specific syntax.

For Pubmed, the search strategy was as follows: ((“oral lichen planus” [Title/Abstract]) OR (“oral lichenoid lesion” [Title/Abstract]) OR (“oral lichenoid reaction” [Title/Abstract])) AND ((“child” [Title/Abstract]) OR (“children” [Title/Abstract]) OR (“pediatric” [Title/Abstract]) OR (“paediatric” [Title/Abstract]) OR (“juvenile” [Title/Abstract]) OR (“adolescent” [Title/Abstract]) OR (“infant” [Title/Abstract])).

For Scopus, the corresponding search strategy was as follows: TITLE-ABS-KEY (“oral lichen planus” OR “oral lichenoid lesion” OR “oral lichenoid reaction”) AND TITLE-ABS-KEY (child OR children OR pediatric OR paediatric OR juvenile OR adolescent OR infant).

For the Cochrane Library, the following search strategy was used:

(“oral lichen planus” OR “oral lichenoid lesion” OR “oral lichenoid reaction”) AND (child OR children OR pediatric OR paediatric OR juvenile OR adolescent OR infant).

After the application of publication date, language, age, and relevance filters, 13 studies were included in the final review.

The inclusion criteria comprised studies involving pediatric patients younger than 18 years of age with a diagnosis of OLP or OLL. Only English-language publications containing original clinical data, including case reports, case series, retrospective studies, and observational studies, were considered eligible. Studies were excluded if they focused exclusively on adult populations, were published in languages other than English, did not provide adequate clinical information for analysis, or were review articles lacking original patient data. Data extracted from each study included patient demographics, lesion location, clinical presentation, histopathological findings, treatment modalities, and outcomes.

## 3. Results

### 3.1. Study Characteristics

Thirteen studies met the inclusion criteria and were included in the final review (Table 1 and Table 2). The studies consisted primarily of case reports describing pediatric patients with OLP. A limited number of reports also described OLL. These reports are discussed separately because of their diagnostic overlap with OLP. Patient ages ranged from 3 to 16 years, with both sexes being represented. The buccal mucosa was the most frequently involved oral site, followed by the tongue, lips, gingiva, vestibular mucosa, and retromolar region. While most patients presented with isolated oral lesions, several exhibited extraoral manifestations involving the skin, genital mucosa, nails, or other mucosal surfaces. The dataset also demonstrated considerable clinical heterogeneity, including reticular, papular, plaque-like, erosive, atrophic, and bullous variants of OLP [28,29,30].

Liu & Yang (2025) [9] reported that the patient was a young child who was unwilling to undergo a biopsy, and the clinical diagnosis of OLP was made based on the characteristic clinical symptoms. Bastos et al. (2016) performed a biopsy, but the final diagnosis was oral lichenoid reaction. Soares & Mendonça (2016) [10] performed a biopsy, but the final diagnosis was oral drug-induced lichenoid reaction. Spirito et al. (2023) [25] did not report histopathological findings. Diagnosis was based on clinical findings and medical history, as histopathological examination was performed only when considered useful for differential diagnosis. Finally, Shikha et al. (2022) [26] reported that a biopsy was not performed in one patient because the patient’s family did not provide consent for the procedure (Table 3).

### 3.2. Epidemiology and Etiopathogenesis

While OLP appears to be prevalent in adults, with a pooled worldwide prevalence of 0.89–1.01%, pediatric disease is extremely rare, with a prevalence of about 0.03% [29,31,32]. OLP in adults mostly affects women and people older than 40 years of age, whereas in children, there is a more equal distribution between sexes, and the condition usually occurs in late childhood and early adolescence, at an average age of about 11 years [29,31]. In addition, the vast majority of published pediatric cases come from Asia, especially India [29]. Recent data indicate that immune mechanisms play a major role in OLP development via the cytotoxicity of T cells against basal keratinocytes. There is also a genetic predisposition to develop OLP, as demonstrated in a family case presented by Wang et al. [12].

There have been many cases wherein temporal associations between possible triggers and onset of the disease have been noted [8]. For instance, Lahouel et al. noted a case of severe childhood lichen planus pemphigoides following the hepatitis A vaccine, but no causality could be established [8]. Presently, there is no clear data available to suggest that such findings must be viewed carefully since most findings are based on case reports alone [8,23]. Brufau-Cochs et al. reported severe OLP that ultimately revealed underlying X-linked lymphoproliferative disease type 1 [26]. Additional reports described OLL associated with chronic lollipop use [25] and recombinant human growth hormone therapy in a patient with Turner syndrome [10].

### 3.3. Clinical Presentation

The reticular form was the most frequently reported clinical presentation. Typical lesions appeared as bilateral white interlacing striae involving the buccal mucosa, tongue, gingiva, and vestibular mucosa [1,2,3,12].

A seven-year-old girl described by Chinnasamy et al. presented with multiple white papular lesions exhibiting radiating white lines affecting both buccal mucosae and the upper labial mucosa [1]. Sharma et al. reported bilateral grayish-white patches surrounded by radiating white striae involving the gingiva and vestibular mucosa [3]. Hasan et al. described classic bilateral Wickham’s striae involving the buccal mucosa and tongue in an eight-year-old boy [2].

An erosive variant was reported by Liu and Yang in a nine-year-old boy with β-thalassemia major who had previously undergone bone marrow transplantation [9]. A linear variant extending from the skin of the upper lip into the oral cavity was described by Thomas and Betsy [9].

Two reports documented severe multisystem disease involving both oral and extraoral tissues [8,26].

Most reticular lesions were asymptomatic and discovered during routine oral examinations. Symptomatic cases were generally associated with erosive disease and manifested as burning sensation, pain, or discomfort during eating [2,9].

The more extensive case series reported by Spirito et al. reinforced the fact that reticular type still predominates in pediatric cases, but lesions such as plaques, papules, atrophy, erosion, and vesicles were also noted [29]. The tongue, especially the dorsum along with the bilateral buccal mucosa, is the most common site for lesions. Some patients had multiple oral sites involved, like the tongue, buccal mucosa, palate, gingiva, and lingual margin [29,30].

Pigmentation associated with reticular lesions was observed in several patients from the Shikha et al. series [30], whereas Gamal-AbdelNaser et al. described an unusual butterfly-shaped ulcer on the tongue associated with bilateral buccal melanotic pigmentation and pseudo-lymphomatous histopathological changes [28]. These observations further expand the spectrum of clinical manifestations reported in pediatric OLP [28,30].

### 3.4. Histopathological Findings

Histopathological examination was performed in most studies and consistently demonstrated findings characteristic of OLP. Common microscopic features included hyperkeratosis or parakeratosis, acanthosis, basal cell degeneration, saw-tooth rete ridges, and a dense subepithelial band-like lymphocytic infiltrate [1,2,3,11,12,26]. Additional data from the large pediatric case series demonstrated the persistence of these features in terms of their histopathology. Basal cell degeneration, hyperkeratosis/parakeratosis, Civatte bodies, acanthosis, and dense lymphocytic band-like infiltrates were observed in many patients [29,30]. Less common observations included papillomatosis, mild epithelial dysplasia, melanin incontinence, and subepithelial clefting [29,30]. Epithelial dysplasia may coexist with OLP; they are not mutually exclusive, since OLP constitutes an oral potentially malignant disorder [33]. Regarding the other observations, a possible explanation is that either OLP was present in terms of a systemic autoimmune disease, yet undiagnosed, at the time of the publication or that OLP coexisted with another mucocutaneous autoimmune disease. For example, melanin incontinence is a microscopic finding where melanin pigment drops out of the epidermis and accumulates in the dermis. It happens when inflammation damages the lower layer of the epidermis, causing immune cells called macrophages to swallow the loose pigment. This is possible in both lichen planus and lupus erythematosus. Subepithelial clefting is a microscopic finding where the top layer of tissue (epithelium) separates from the lower layer (connective tissue). This is noticed in autoimmune blistering diseases like mucous membrane pemphigoid or bullous pemphigoid. It would be possible as well, in a lichen planus pemphigoides case. Papillomatosis and lichen planus may also occasionally coexist.

Although characteristic clinical findings may strongly suggest OLP, histopathological examination is recommended to support the diagnosis and exclude other oral mucosal disorders. The final diagnosis should be established through clinicopathological correlation rather than histopathological findings alone [34,35,36].

These histopathological findings are characteristic of OLP but are not entirely specific, as similar features may also be observed in OLL and other inflammatory mucosal disorders [34,35,36,37,38]. Histopathological assessment is particularly important because several disorders may mimic OLP clinically [36]. Therefore, clinicopathological correlation remains essential for establishing an accurate diagnosis [34,35,36,38]. Conditions such as oral lichenoid reactions, lupus erythematosus, graft-versus-host disease, and chronic ulcerative disorders may exhibit similar clinical features [34,39]. Therefore, clinicopathological correlation remains fundamental for establishing an accurate diagnosis [34]. The essential nature of a biopsy is further highlighted by the clinical data showing how frequently pediatric presentations mask or overlap with other complex systemic pathologies. For instance, a biopsy is often the deciding factor in distinguishing true idiopathic OLP from OLL induced by hypersensitivity or localized mechanical trauma, as illustrated in the case of chronic lollipop use [25] or drug-induced reactions [10]. Clinicians must remain highly vigilant, as severe mucocutaneous manifestations can serve as an early clinical marker for severe underlying systemic issues or primary immunodeficiencies, such as X-linked lymphoproliferative disease type 1 [26] or post-transplantation complications in patients with ß-thalassemia major [9]. Relying solely on macroscopic features like Wickham’s striae without a confirmatory tissue biopsy creates an unacceptable risk of diagnostic delay or misclassification, which can ultimately compromise patient care in these vulnerable young cohorts.

### 3.5. Treatment and Outcomes

Topical corticosteroids represented the most frequently prescribed treatment modality and were associated with favorable outcomes in most patients [1,2,3].

Most patients receiving therapy with topical corticosteroids showed improvement or total recovery clinically after several weeks to months. However, asymptomatic reticular lesions were treated non-surgically by follow-up and observation at intervals, while others had spontaneous resolution of the lesions. The systemic use of corticosteroids was only used in cases with systemic disease [29,30].

Despite the good results that were achieved with treatments, some of the patients had recurrence, incomplete or unresolved lesions, and some even had persisting lesions even after treatment [28,29,30]. This is an indication of the recurrent nature of the pediatric OLP. Loss to follow-up was also noted among some of the patients [28,29,30].

Chinnasamy et al. reported significant clinical improvement following treatment with topical triamcinolone acetonide [1]. Sharma et al. achieved lesion resolution using topical corticosteroids followed by aloe vera gel [3]. Hasan et al. observed near-complete resolution after treatment with steroid mouth rinses [2].

The erosive case reported by Liu and Yang responded favorably to intralesional triamcinolone injections [9]. Thomas and Betsy documented satisfactory improvement with topical tacrolimus [11].

Systemic prednisone was highly effective in treating the severe mucocutaneous case secondary to vaccination described by Lahouel et al. [8]. Separately, the patient with underlying X-linked lymphoproliferative disease type 1 described by Brufau-Cochs et al. required aggressive systemic management with cyclosporine, acitretin, and ultimate hematopoietic stem cell transplantation to achieve complete remission [26].

The importance of trigger identification was highlighted by the oral lichenoid reactions reported by Bastos et al. [25] and Soares and Mendonca [10]. In both cases, clinical improvement occurred following elimination or management of the precipitating factor.

Recent studies further prove the use of topical steroids as the first-line treatment modality in symptomatic OLP due to their effective action in terms of relieving pain, redness, and severity of lesions [40]. Topical high-potency steroids continue to be used as the standard therapy in lichen planus, while topical calcineurin inhibitors, such as tacrolimus, can be applied to patients with resistant disease or those who cannot tolerate steroid therapy [41]. Moreover, innovative treatment modalities, such as photobiomodulation, have shown positive outcomes in adults, but no sufficient research is available regarding the treatment of pediatrics [42,43]. Consequently, treatment decisions in children should be individualized according to lesion severity, symptom burden, and careful long-term clinical follow-up [40].

## 4. Discussion

The present review highlights the rarity of OLP in childhood and confirms that the available literature remains limited primarily to isolated case reports. Despite the limited evidence base, several important observations emerge.

The findings suggest that pediatric OLP shares many clinical and histopathological characteristics with adult disease [4,44]. In both populations, the reticular form represents the most common clinical subtype [44]. Likewise, the buccal mucosa remains the site most frequently affected [44]. However, compared with adults, children appear to present less frequently with erosive disease and generally demonstrate a more favorable response to treatment [4]. The rarity of pediatric OLP may reflect differences in immune function, environmental exposure, or genetic susceptibility during childhood [4]. Previous pediatric reviews have similarly emphasized the uncommon nature of the disease and the limited availability of long-term follow-up data in children [6,7].

The rarity of pediatric OLP may also contribute to delayed diagnosis, as clinicians are generally less likely to include OLP in the differential diagnosis of persistent oral white lesions in children. Furthermore, many pediatric patients present with asymptomatic reticular lesions that are detected only during routine dental examinations. Consequently, increased awareness among pediatric dentists, oral medicine specialists, dermatologists, and general practitioners is essential to facilitate early recognition and timely management [1,3,6,7].

The reticular subtype appears to be the most common presentation in pediatric patients, closely resembling adult disease [1,2,3,12]. Bilateral involvement of the buccal mucosa remains the hallmark clinical feature. However, clinicians should be aware that erosive, linear, familial, and severe multisystem forms may also occur [8,9,11,12,26].

This is further bolstered by the presence of larger case series in pediatric populations [29,30]. Among nearly two dozen pediatric cases, reticular type lesions prevailed, but plaque, papules, atrophic, erosive, and bullous types were seen to be less common. Also, bilateral occurrence on buccal mucosa and dorsal tongue remained to be the most frequent anatomic distribution, thus reaffirming prior findings that pediatric OLP is quite similar to adult OLP but significantly less common [29,30].

The broader spectrum of clinical presentations identified in the larger pediatric case series also highlights the importance of performing a comprehensive oral examination [9,11,29,30]. Although bilateral reticular lesions remain the characteristic presentation, clinicians should recognize that pediatric OLP may manifest as plaque-like, papular, erosive, atrophic, bullous, or linear lesions affecting multiple oral sites simultaneously. Awareness of this clinical variability may reduce misdiagnosis and facilitate earlier recognition of the disease [9,11,29,30].

Histopathological findings were remarkably consistent among studies and strongly support the diagnosis of an immune-mediated disorder [1,2,3,11,12,26]. The repeated observation of basal cell degeneration and dense subepithelial lymphocytic infiltration reinforces the value of biopsy as part of the diagnostic work-up [34,37]. However, current recommendations emphasize that the diagnosis of OLP should be based on the integration of clinical and histopathological findings [34,35,36,38,45]. Current diagnostic recommendations emphasize the integration of both clinical and histopathological criteria, as clinicopathological discrepancies have been reported when diagnosis relies solely on clinical presentation [34,39].

The reviewed studies also demonstrate that pediatric OLP may be associated with genetic predisposition, systemic diseases, medications, environmental exposures, and immune dysregulation [8,10,12,25,26]. These associations emphasize the importance of obtaining a comprehensive medical history and considering potential precipitating factors when evaluating pediatric patients with oral white lesions. Contemporary evidence supports multifactorial pathogenesis involving antigen-specific cell-mediated immune responses, activation of cytotoxic CD8+ T lymphocytes, cytokine dysregulation, and epithelial apoptosis [13,14,15,17,18,20]. In addition, oxidative stress has been proposed as a contributing factor, with increased oxidative damage and altered antioxidant defenses reported in patients with OLP [19].

Topical corticosteroids remain the first-line treatment owing to their anti-inflammatory and immunosuppressive properties [46,47] and are the cornerstone of treatment, appearing effective for most children [1,2,3]. More aggressive therapies should be reserved for severe, refractory, or syndromic cases [8,26]. Importantly, trigger elimination may be curative in cases of oral lichenoid reactions [10,25]. Calcineurin inhibitors such as tacrolimus may be considered in refractory cases [47]. Systemic corticosteroids and immunomodulatory agents should generally be reserved for severe, extensive, or treatment-resistant disease [47]. The favorable outcomes observed in the studies included in this review suggest that most pediatric cases can be successfully managed with conservative therapy and regular follow-up [46]. Furthermore, the success of non-pharmacological interventions in specific pediatric cohorts highlights the clinical need to distinguish between true idiopathic OLP and transient oral lichenoid reactions. Although topical corticosteroids were effective in most patients, the available evidence also indicates that treatment should be individualized according to disease severity, lesion distribution, symptom burden, and the presence of associated systemic conditions [29,30,46,47]. While symptomatic erosive lesions generally require pharmacological intervention, asymptomatic reticular lesions may often be managed conservatively with periodic clinical follow-up. When managing pediatric oral mucosal lesions, local environmental irritants, secondary hypersensitivities, and dietary habits must be exhaustively investigated alongside standard medical therapy. Identifying and eliminating a simple behavioral trigger can occasionally result in complete, permanent resolution without requiring prolonged topical immunosuppressive courses [25]. This distinction is critical because long-term, continuous exposure to potent corticosteroids or calcineurin inhibitors should be minimized in young children whenever possible to prevent localized or systemic adverse effects [46,47].

Interestingly, some patients with asymptomatic disease were able to do without medication while remaining stable or improving spontaneously. This fact indicates that close clinical observation may be a reasonable therapeutic approach in a certain number of children who have asymptomatic reticular disease [29,30].

Despite topical corticosteroids being the most common treatment option for symptomatic OLP, the use of systemic corticosteroids becomes necessary in those patients who suffer from extensive or more severe OLP. However, prolonged use of corticosteroids leads to many adverse effects like oral candidiasis, mucosal atrophy, impaired wound healing, and such metabolic disorders as osteoporosis, hypertension, adrenal suppression in the case of systemic administration of corticosteroids [48]. Thus, there is a need for other safe and effective treatment options. In recent times, photodynamic therapy (PDT) has become an interesting treatment option for some OLP patients [48]. It involves local administration of a photosensitizer followed by its activation with light and subsequent production of reactive oxygen species with anti-inflammatory and immunomodulating properties [48]. The benefits of using PDT in the treatment of OLP include its minimally invasive character, safety, good cosmetic results, and the ability to repeat the treatment procedure multiple times. However, despite the promising clinical outcomes observed after photodynamic treatment, there is still a lack of evidence about its efficacy due to the few existing studies and different treatment protocols [48].

Although most pediatric patients demonstrated favorable clinical outcomes, several reports documented recurrent disease, persistent lesions, incomplete resolution, or loss to follow-up. These findings emphasize that pediatric OLP should be regarded as a chronic inflammatory disorder requiring long-term clinical surveillance even after apparent clinical improvement [7,28,29].

Although no cases of malignant transformation were identified among the pediatric patients included in this review, OLP is currently classified as an oral potentially malignant disorder [27]. Chronic inflammation and persistent epithelial injury have led some authors to consider OLP a preneoplastic inflammatory condition [16]. According to recent systematic reviews and meta-analyses in adults, the rate of malignancy is found to be between 0.9% and 1.4%, while in the few pediatric case reports included in this review, there has been no evidence of such malignancies [5,32,49]. While this risk appears extremely low or practically non-existent in children based on short-term data, meticulous long-term clinical surveillance remains highly advisable because of the inherently chronic nature of the disease and the notable scarcity of long-term pediatric outcome data [6,7].

### Limitations

The principal limitation of this review is the limited number of available studies and the predominance of case reports, which limits the strength of evidence. Larger multicenter studies are needed to improve understanding of disease pathogenesis, natural history, and treatment outcomes. Additionally, the screening process itself highlights significant publication bias, as the current literature is heavily dominated by isolated case reports rather than multi-center observational cohorts. This lack of standardized epidemiological reporting restricts our ability to draw generalized conclusions regarding the precise global prevalence or gender predilection of OLP in younger age brackets. Furthermore, another limitation is the lack of a literature search in Web of Science. Future research should focus on clarifying the immunological and molecular mechanisms involved in pediatric OLP [14,17,20] and on establishing evidence-based management protocols specifically tailored to children [6,7].

## 5. Conclusions

OLP is an uncommon but clinically significant inflammatory oral mucosal disorder in children. The reticular subtype represents the most common clinical presentation, although erosive, linear, familial, and severe mucocutaneous variants have also been reported. Histopathological examination remains an important diagnostic tool and typically reveals basal cell degeneration with a dense band-like lymphocytic infiltrate. Current evidence supports an autoimmune response involving antigen-presenting cells and regulatory T-lymphocytes, probably triggered by keratinocytes.

Topical corticosteroids remain the first-line therapeutic approach and are associated with favorable outcomes in most patients. Increased awareness among dentists, pediatricians, dermatologists, and oral medicine specialists is essential for timely diagnosis and appropriate management. Future multicenter studies are needed to establish evidence-based guidelines and further clarify the pathogenesis and optimal treatment strategies for pediatric OLP.

## Figures and Tables

**Table 1:** Summary of the included studies as well as their key findings presented as a study-level table.

Study	Age/Sex	Site	Clinical Presentation	Histopathological Findings	Treatment	Outcome
Chinnasamy et al. [1]	7/F	Bilateral buccal and upper labial mucosa	Multiple white papular lesions with radiating lines (reticular OLP)	Parakeratotic stratified squamous epithelium with basal cell degeneration	Topical triamcinolone acetonide 0.1%	Complete relief of symptoms and reduction in lesion size after 3 months
Sharma et al. [3]	12/F	Gingiva and vestibular mucosa	Grayish-white patches with peripheral radiating white striae	Hyperplastic parakeratinized epithelium, acanthosis, basal cell degeneration and band-like lymphocytic infiltrate	Topical corticosteroid followed by aloe vera gel	Complete clinical resolution within one month
Hasan et al. [2]	8/M	Buccal mucosa and tongue	Bilateral Wickham’s striae with a 2 cm × 3 cm erosive lesion on the left buccal mucosa and tongue depapillation	Acanthosis with dense band-like lymphocytic infiltrate	Steroid mouth rinse	Marked resolution after 15 days
Wang et al. [12]	3/M	Lips, tongue and bilateral buccal mucosa	Diffuse reticular and papular lesions with erosive areas. Familial History	Histopathological features compatible with OLP	Recombinant bovine basic fibroblast growth factor	Lesions gradually resolved. 8 year long-term follow-up
Liu and Yang [9]	9/M	Lips, tongue and buccal mucosa	Erosive lesions with yellowish-white pseudomembrane. History of β-Thalassemia major and bone marrow transplant	Biopsy not performed	Intralesional triamcinolone and supportive therapy	Significant symptom improvement
Thomas and Betsy [11]	14/M	Upper lip and oral mucosa	Linear hyperkeratotic plaque extending from skin to oral mucosa	Marked parakeratosis, basal cell degeneration, colloid bodies and dense lymphocytic infiltrate	Topical tacrolimus 0.1%	Good clinical improvement
Lahouel et al. [8]	8/F	Oral, nasal and vulvar mucosa with widespread skin involvement	Violaceous papules, plaques and bullous lesions	Subepidermal blister with inflammatory infiltrate	Systemic prednisone	Rapid improvement within 10 days
Brufau-Cochs et al. [26]	14/M	Lips, buccal mucosa and tongue	Extensive reticular lesions and mucosal desquamation	Interface mucositis with hyperkeratosis and dense lymphocytic infiltrate	Cyclosporine, acitretin and hematopoietic stem cell transplantation	Complete remission
Bastos et al. [25]	15/F	Tongue and buccal mucosa	White plaques consistent with oral lichenoid reaction	Histopathological findings suggestive of OLP	Topical corticosteroids (failed), elimination of lollipop habit	Resolution following removal of trigger
Soares and Mendonca [10]	9/F	Labial and buccal mucosa, palms and soles	Interlacing white keratotic lines and scaly papules	Band-like lymphocytic infiltrate at the epithelial-connective tissue interface	Clobetasol propionate and dexamethasone mouthwash	Significant improvement after one month
Gamal-AbdelNaser et al. [28]	8/M	Tongue, bilateral buccal mucosa	Butterfly-shaped superficial ulcer surrounded by white radiating lines, erythematous depapillated tongue, bilateral linear melanotic pigmentation of the buccal mucosa	Epithelial atrophy, Civatte bodies, subepithelial clefting, and dense band-like lymphocytic infiltrate with tertiary lymphoid follicle-like arrangement	Topical miconazole followed by topical triamcinolone acetonide 0.1%	Pain relieved after 1 month; recurrent episodes of remission and exacerbation; lost to follow-up
Spirito et al. (Case series, *n* = 13)	6–16 y (7 M/6 F)	Mainly tongue (12/13), buccal mucosa (6/13), palate (1/13)	Predominantly reticular/papular and plaque-like lesions; occasional ulcerative, bullous and atrophic lesions	Typical OLP features with band-like lymphocytic infiltrate and basal cell degeneration	Mainly topical corticosteroids; observation in asymptomatic cases; systemic steroids in one patient	Complete/partial remission in most patients; recurrences or persistent lesions in some cases; no malignant transformation reported.
Shikha et al. (Case series, *n* = 6)	11–13 years (5 M, 1 F)	Predominantly bilateral buccal mucosa and tongue; gingiva and retromolar region in some cases	Mainly reticular OLP with plaque-like lesions; some cases presented with erythema and pigmentation; one patient was asymptomatic	Histopathological findings, when performed, were consistent with OLP, including hyperkeratosis/parakeratosis, basal cell degeneration, band-like lymphocytic infiltrate, and saw-tooth rete ridges; one patient was diagnosed clinically without biopsy	Topical triamcinolone acetonide 0.1% in symptomatic patients; regular follow-up without treatment for asymptomatic cases	Marked clinical improvement in treated patients, spontaneous improvement in one asymptomatic case, and loss to follow-up in two patients.

**Table 2:** Summary of the included studies as well as their key findings presented as a patient-level table.

Study	Age/Sex	Site	Clinical Presentation	Histopathological Findings	Treatment	Outcome
Chinnasamy et al. [1]	7/F	Bilateral buccal and upper labial mucosa	Multiple white papular lesions with radiating lines (reticular OLP)	Parakeratotic stratified squamous epithelium with basal cell degeneration	Topical triamcinolone acetonide 0.1%	Complete relief of symptoms and reduction in lesion size after 3 months
Sharma et al. [3]	12/F	Gingiva and vestibular mucosa	Grayish-white patches with peripheral radiating white striae	Hyperplastic parakeratinized epithelium, acanthosis, basal cell degeneration and band-like lymphocytic infiltrate	Topical corticosteroid followed by aloe vera gel	Complete clinical resolution within one month
Hasan et al. [2]	8/M	Buccal mucosa and tongue	Bilateral Wickham’s striae with a 2 cm × 3 cm erosive lesion on the left buccal mucosa and tongue depapillation	Acanthosis with dense band-like lymphocytic infiltrate	Steroid mouth rinse	Marked resolution after 15 days
Wang et al. [12]	3/M	Lips, tongue and bilateral buccal mucosa	Diffuse reticular and papular lesions with erosive areas. Familial History	Histopathological features compatible with OLP	Recombinant bovine basic fibroblast growth factor	Lesions gradually resolved. 8 year long-term follow-up
Liu and Yang [9]	9/M	Lips, tongue and buccal mucosa	Erosive lesions with yellowish-white pseudomembrane. History of β-Thalassemia major and bone marrow transplant	Biopsy not performed	Intralesional triamcinolone and supportive therapy	Significant symptom improvement
Thomas and Betsy [11]	14/M	Upper lip and oral mucosa	Linear hyperkeratotic plaque extending from skin to oral mucosa	Marked parakeratosis, basal cell degeneration, colloid bodies and dense lymphocytic infiltrate	Topical tacrolimus 0.1%	Good clinical improvement
Lahouel et al. [8]	8/F	Oral, nasal and vulvar mucosa with widespread skin involvement	Violaceous papules, plaques and bullous lesions	Subepidermal blister with inflammatory infiltrate	Systemic prednisone	Rapid improvement within 10 days
Brufau-Cochs et al. [22]	14/M	Lips, buccal mucosa and tongue	Extensive reticular lesions and mucosal desquamation	Interface mucositis with hyperkeratosis and dense lymphocytic infiltrate	Cyclosporine, acitretin and hematopoietic stem cell transplantation	Complete remission
Bastos et al. [21]	15/F	Tongue and buccal mucosa	White plaques consistent with oral lichenoid reaction	Histopathological findings suggestive of OLP	Topical corticosteroids (failed), elimination of lollipop habit	Resolution following removal of trigger
Soares and Mendonca [10]	9/F	Labial and buccal mucosa, palms and soles	Interlacing white keratotic lines and scaly papules	Band-like lymphocytic infiltrate at the epithelial-connective tissue interface	Clobetasol propionate and dexamethasone mouthwash	Significant improvement after one month
Gamal-AbdelNaser et al. [24]	8/M	Tongue, bilateral buccal mucosa	Butterfly-shaped superficial ulcer surrounded by white radiating lines, erythematous depapillated tongue, bilateral linear melanotic pigmentation of the buccal mucosa	Epithelial atrophy, Civatte bodies, subepithelial clefting, and dense band-like lymphocytic infiltrate with tertiary lymphoid follicle-like arrangement	Topical miconazole followed by topical triamcinolone acetonide 0.1%	Pain relieved after 1 month; recurrent episodes of remission and exacerbation; lost to follow-up
Spirito et al. [25]	6/F	Bilateral buccal mucosae	Papules with reticular pattern	Focal hyperkeratosis, irregular acanthosis, basal cell liquefaction, and dense band-like lymphocytic infiltrate	Topical corticosteroids	Symptom remission after 5 weeks
	13/F	Dorsum tongue	Plaque lesion	Hyperkeratosis, hypergranulosis, Civatte bodies, basal cell vacuolar degeneration, and band-like inflammatory infiltrate	Topical corticosteroids	Symptom remission after 2 weeks with two acute recurrences
	10/M	Dorsum tongue	Ulcer surrounded by a thin white patch with reticulated borders	Dense lymphocytic infiltrate, basal cell degeneration, moderate acanthosis, and papillomatosis	Topical corticosteroids	Symptom remission after 3 weeks
	13/M	Dorsum tongue and lingual margin	Papules with reticular and plaque-like pattern	Hyperkeratotic epithelium with eosinophilic colloid (Civatte) bodies in the lower epithelium and superficial connective tissue	Topical corticosteroids	Symptom remission after 6 weeks
	14/F	Dorsum tongue, bilateral buccal mucosae, and palate	Papules with reticular pattern	NR (not reported)	Systemic corticosteroids (prednisone 0.25 mg/kg/day) for concomitant juvenile rheumatoid arthritis	Reduction in the extent of lesions
	10/M	Lingual margin and bilateral buccal mucosae	Papules with reticular pattern	NR (not reported)	None	Reduction in the extent of lesions
	6/M	Dorsum tongue	Blisters and plaques	NR (not reported)	Topical corticosteroids	Symptom remission after 4 weeks
	13/M	Dorsum tongue	Papules	Hyperorthokeratinized stratified squamous epithelium with mild dysplasia	Topical corticosteroids	No reduction in lesions
	16/M	Dorsum tongue	Plaque lesion	Hyperplastic squamous epithelium, acanthosis, hyperparakeratosis, basal cell vacuolar degeneration, intraepithelial lymphocytic exocytosis, and band-like subepithelial lichenoid inflammatory infiltrate	None	No reduction in lesions
	15/F	Dorsum tongue and bilateral buccal mucosae	Reticular papules on the buccal mucosa and plaque lesion on the dorsum of the tongue	Thickened squamous epithelium with focal papillomatosis, acanthosis, parakeratosis, mild dysplasia, and subepithelial lymphocytic infiltrate with fibrosis and vascular neoformation	Topical corticosteroids	Partial symptom remission after 4 weeks
	16/F	Ventral tongue and bilateral buccal mucosae	Papules and erythematous lesions	NR (not reported)	None	No reduction in lesions
	15/M	Dorsum tongue and lingual margin	Atrophic lesions and papules with reticular pattern	NR (not reported)	Topical corticosteroids	Symptom remission after 6 weeks
	12/F	Dorsum tongue and bilateral buccal mucosae	Papules	Stratified squamous epithelium with papillomatosis, acanthosis, parakeratosis, and subepithelial lymphocytic infiltrate	None	No reduction in lesions
Shikha et al. [26]	12/M	Bilateral buccal mucosa and anterior dorsal tongue	Bilateral plaque-like white lesions on the buccal mucosa with reticular striations and a white plaque with reticular striations on the anterior dorsum of the tongue	NR (biopsy not performed)	None (regular follow-up)	Marked resolution of lesions on the buccal mucosa and dorsum of the tongue during follow-up
	12/M	Bilateral buccal mucosa, retromolar region, dorsum tongue	Reticular white striae with erythema (buccal mucosa); white plaque on dorsum tongue	Hyperorthokeratosis, basal cell degeneration, indistinct basement membrane, band-like inflammatory infiltrate	Topical triamcinolone acetonide 0.1%	Symptomatic relief; marked lesion resolution, partial tongue resolution
	11/M	Bilateral buccal mucosa, anterior dorsal tongue, labial gingiva	Reticular white striae with pigmentation (buccal mucosa); white patch (tongue); gingival involvement	Hyperkeratosis, basal cell degeneration, lymphocytic and macrophage infiltrate	None (regular follow-up)	Lost to follow-up
	12/M	Bilateral buccal mucosa and buccal vestibule	Bilateral reticular white striae	Parakeratosis, saw-tooth rete ridges, basal cell degeneration, and band-like lymphohistiocytic infiltrate	Topical triamcinolone acetonide 0.1%	Marked lesion resolution after 6 months
	13/M	Bilateral buccal mucosa	Reticular white striae with erythema and pigmentation	Basal cell degeneration, band-like lymphocytic infiltrate, and mild melanin incontinence	Topical triamcinolone acetonide 0.1%	Marked resolution of erythema (right buccal mucosa) and complete resolution of the left buccal lesion after 6 months
	12/F	Bilateral buccal mucosa	Diffuse white papules with peripheral reticular striae	Features consistent with OLP (histopathological confirmation)	Topical triamcinolone acetonide 0.1%	Lost to follow-up

**Table 3:** The presence of histopathological confirmation in the included studies.

Study	Histopathological Confirmation
Chinnasamy et al., 2020 [1]	+ (1/1)
Sharma et al., 2017 [3]	+ (1/1)
Hasan et al., 2020 [2]	+ (1/1)
Wang et al., 2020 [12]	+ (1/1)
Liu & Yang, 2025 [9]	− (0/1)
Thomas & Betsy, 2018 [11]	+ (1/1)
Lahouel et al., 2022 [8]	+ (1/1)
Brufau-Cochs et al., 2026 [26]	+ (1/1)
Bastos et al., 2016 [25]	+ (1/1)
Soares & Mendonça, 2016 [10]	+ (1/1)
Gamal-AbdelNaser, 2023 [28]	+ (1/1)
Spirito et al., 2023 [29]	8+/5− (NR)
Shikha et al., 2022 [30]	5+/1−

+ indicates that biopsy and histopathological examination were performed; − indicates that biopsy was not performed; NR indicates that histopathological findings were not reported in the original study.

## Data Availability

The original contributions presented in this study are included in the article. Further inquiries can be directed to the corresponding author.
